# Dissecting hair breakage in alopecia areata: the central role of dysregulated cysteine homeostasis

**DOI:** 10.1007/s00726-024-03395-5

**Published:** 2024-05-21

**Authors:** Wen Xu, Bo Xie, Dongfan Wei, Xiuzu Song

**Affiliations:** 1 School of Medicine, Zhejiang University, Yuhangtang Rd 866, Hangzhou, 310009 People’s Republic of China; 2 Department of Dermatology, Hangzhou Third People’s Hospital, Affiliated Hangzhou Dermatology Hospital, Zhejiang University School of Medicine, Hangzhou Third Hospital, Affiliated to Zhejiang Chinese Medical University, West Lake Ave 38, Hangzhou, 310009 People’s Republic of China

**Keywords:** Alopecia areata, Cysteine metabolism, Hair breakage, Exclamation mark hairs, Ferroptosis

## Abstract

In the initial stages of Alopecia Areata (AA), the predominance of hair breakage or exclamation mark hairs serves as vital indicators of disease activity. These signs are non-invasive and are commonly employed in dermatoscopic examinations. Despite their clinical salience, the underlying etiology precipitating this hair breakage remains largely uncharted territory. Our exhaustive review of the existing literature points to a pivotal role for cysteine—a key amino acid central to hair growth—in these mechanisms. This review will probe and deliberate upon the implications of aberrant cysteine metabolism in the pathogenesis of AA. It will examine the potential intersections of cysteine metabolism with autophagy, ferroptosis, immunity, and psychiatric manifestations associated with AA. Such exploration could illuminate new facets of the disease's pathophysiology, potentially paving the way for innovative therapeutic strategies.

## Introduction

Alopecia areata (AA)–a non-scarring alopecia form–affects approximately 2% of the global population (Lee et al. 2020). AA can range in severity, from patches of hair loss to total loss of hair on the scalp (alopecia totalis) or body (alopecia universalis) (Cordaro et al. 2021). The cyclical and unpredictable nature of relapse and remission (Feritas et al. 2023) significantly affects the patient’s quality of life, often leading to mental health disorders such as anxiety and depression (Lauron et al. 2023a; Okhovat et al. 2023). Although the pathogenesis of AA is understood to involve disruption of the hair follicle immune privilege (Mo et al. 2020) and oxidative stress (Shakoei et al. 2023b; Peterle et al. 2023), the exact etiology remains unclarified. Understanding the root causes is crucial as it can guide treatment strategies. Early diagnosis is paramount for mitigating disease progression and lessening its impact on affected individuals.

Recently, a correlation between KRT82 gene mutations and increased susceptibility to AA has been identified, proposing that dysfunction of keratin might compromise the keratinizing layer of the hair follicle in these patients (Erjavec et al. 2022). These mutations appear to prompt the infiltration of CD8+ T cells around hair follicles, triggering subsequent immune responses. This discovery underscores the pivotal role of maintaining keratin integrity in the pathogenesis of AA (Sáurez-Fauriñas et al. 2015; Oka et al. 2020). Studies have suggested that this integrity depends on both the content and metabolic state of its cysteine (Erlich et al. 2020; Feroz et al. 2020). This prompts us to reassess the relationship between AA hair characteristics and cysteine metabolism. 

**Fig. 1:**
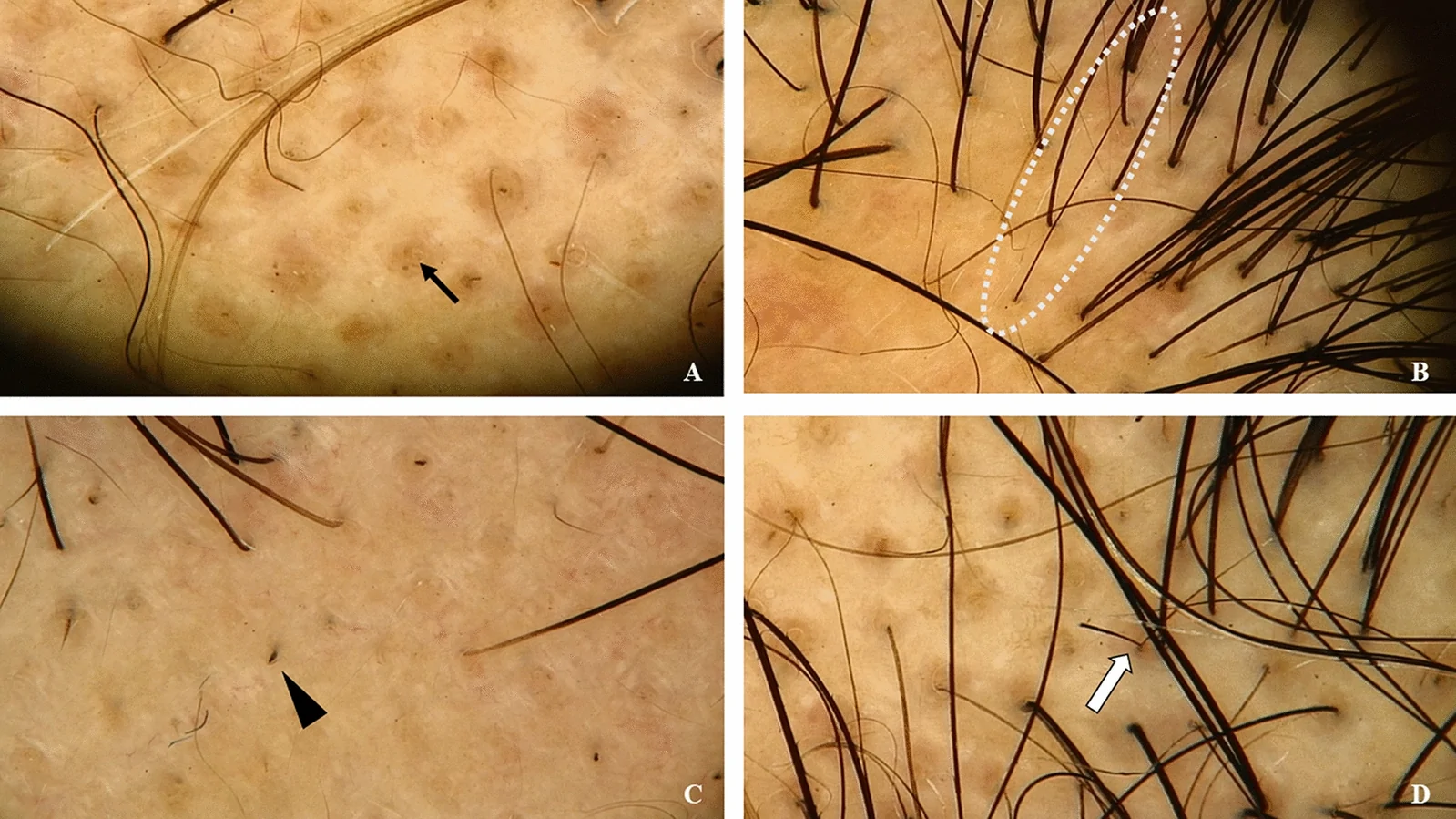
Trichoscopic features of AA. **A** Yellow Dot: Black arrow,  × 20. These dots are common in AA’s stationary phase, resulting from oxidation in hair follicles. **B** Erect and Regenerated Hairs: Dashed circle, × 20. Seen in AA’s recovery phase, these upright hairs often follow medication or spontaneous healing, and have a small cone at the tip. **C** Black Dot: Black triangle, × 20. Common in AA's active phase, they mark hair breakage at the scalp. **D** Exclamation Mark Hair: White arrow, × 20. Indicative of AA worsening, it features a thin proximal end

Several distinctive features of AA can be identified through trichoscopic examination. These features include yellow dots, erect and regenerated hairs, black dots, and exclamation mark hairs (Waśkiel et al. 2018; Gómez-Quispe et al. 2023) (Fig. 1). Notably, the latter two are frequently recognized as early indicators of AA activity (Alsenaid et al. 2023). Furthermore, they serve as markers of either existing or impending hair breakage in the context of AA. Nevertheless, the exact causes of these trichoscopic signs and their pathophysiological significance remain under active investigation. As the hair shaft primarily comprises keratin, enriched in cysteine, we postulate that these trichoscopic signs might be manifestations of localized reduction or metabolic disturbances of cysteine in keratin. Such disturbances could potentially lead to decreased disulfide bond formation (Plowman et al. 2021; Ioannidis et al. 2022), thus affecting hair structure stability. Although intriguing, this theory demands further exploration and validation.

**Fig. 2:**
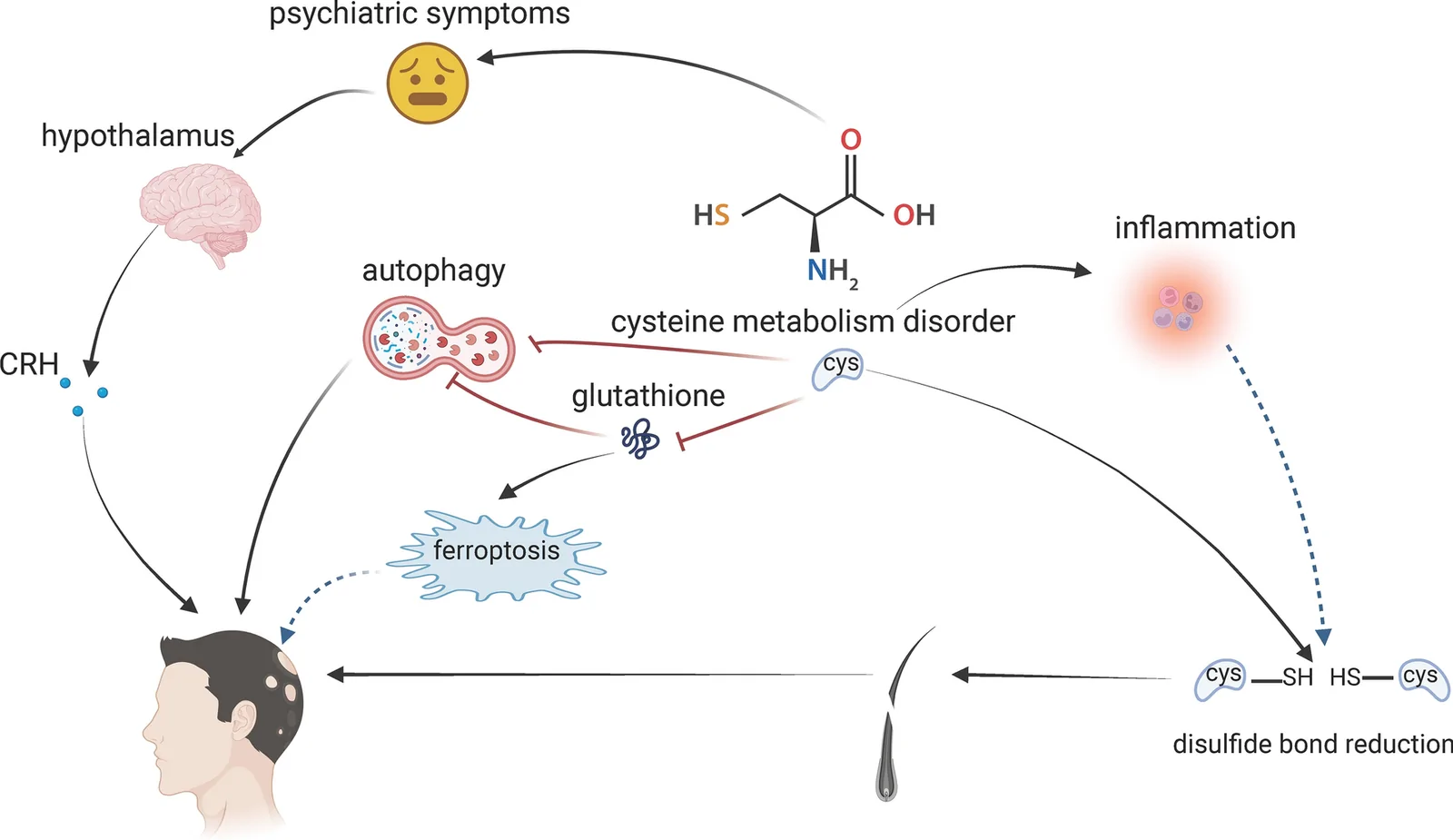
Schematic overview

Expanding on these findings, we propose that cysteine metabolism may play a pivotal role in keratin function abnormalities and damage to the hair follicle’s keratinizing layer. In this review, we provide an in-depth examination of this hypothesis, discussing the potential impacts of associated factors such as autophagy, immunity, psychiatric symptoms, and ferroptosis. Pursuing this research trajectory, we also propose new potential therapeutic strategies for AA, primarily focusing on cysteine metabolism modification (Fig. 2).

Arrows denote promotional effects, while T-bars indicate inhibitory actions. Dashed lines accompanied by question marks represent our hypotheses, for which literature support is currently lacking. CRH, corticotropin-releasing hormone. Created with BioRender.com.

## The role of cysteine metabolism in AA: a potential connection

Cysteine, a semi-essential amino acid (Yang et al. 2023) that is abundant in hair, plays a crucial role in maintaining the stability of the hair shaft through its involvement in disulfide bond formation (Theil et al. 2019; Su et al. 2020). Any disruption to this process could potentially lead to hair fragility and subsequent hair breakage or loss.

Cysteine is derived from two primary pathways: endogenous synthesis within the body, and exogenous sources, including dietary intake and pharmaceutical agents such as *N*-acetylcysteine (NAC) (Kožich and Stabler 2020; Bonifácio et al. 2021). Its cellular absorption primarily occurs through three specific membrane transporters: the cysteine–glutamate transporter exchange system (System XC-), the excitatory amino acid transporter 3 (EAAT3), and the alanine–serine–cysteine transporter (ASCT), with ASCT1 and ASCT2 as subtypes (Paul et al. 2018). System XC- plays a significant role by transporting cysteine into cells as cystine, which is then reduced back to cysteine by reducing agents like glutathione (GSH) (Parker et al. 2021). This mechanism is the primary mode of cellular cysteine absorption. Alternatively, EAAT3 can directly assimilate cysteine. However, research suggests that the inhibitory action of glutamate and aspartate on EAAT3 may cause neuronal oxidative damage due to restricted cysteine absorption (Malik et al. 2019). ASCT1 and ASCT2 assist in the transport process of other small and medium neutral amino acids, such as leucine (Freidman et al. 2020). Their function is crucial for maintaining the balance of cysteine within cells, and disturbances in their function can result in health disorders. For instance, glioma cells are reported to heavily rely on upregulated System XC- expression for cysteine uptake, facilitating GSH production and tumor growth (Floros et al. 2021). Therefore, inhibiting System XC- expression in tumor cells could present a potential anti-tumor strategy.

Endogenous cysteine synthesis is predominantly achieved through the conversion of homocysteine (Hcy) and through GSH and protein degradation. Hcy, primarily obtained from methionine as an intermediate metabolite in the methionine cycle, is converted into cystathionine via cystathionine-β-synthase (CBS) and cystathionine-γ-lyase (CSE) (Zatsepina et al. 2020). Studies have shown that systemic CBS gene knockout mice exhibit an increased predisposition towards hair loss and present with significantly higher in vivo oxidative stress indicators than their wild-type counterparts (Nakladal et al. 2022). Moreover, γ-glutamyl transpeptidase (GGT) degrades GSH into glutamic acid and cysteinyl glycine, which are subsequently hydrolyzed into cysteine and glutamate (Fu et al. 2021). This process is particularly active under conditions of low cysteine levels or cellular starvation, suggesting that GSH may act as an intracellular cysteine reservoir. In parallel, intracellular proteins can also be hydrolyzed to produce cysteine, although the amount generated this way is relatively minimal (Spears et al. 2021).

In hair follicles, cysteine predominantly engages in processes encompassing antioxidative stress response, energy transmission, keratin stabilization, protein cysteinylation, and hair pigmentation. As a pivotal precursor in GSH synthesis, cysteine is indispensable for counteracting oxidative stress, primarily by neutralizing reactive oxygen species (ROS) from oxidative stress (Wu et al. 2021) and modulating ferroptosis, an iron-associated cell death (Badgley et al. 2020). Cysteine, as a sulphur donor, contributes its sulphur atom in diverse biosynthetic procedures, transferring it to iron to construct Fe-S clusters (Lill and Freibert 2020). These clusters critically underpin the mitochondrial respiratory chain in hair follicle cells, subsequently promoting ATP synthesis (Lill 2009), which furnishes the requisite energy for hair follicle growth. Within hair structures, the disulphide bonds, a salient component formed by cysteine, are instrumental in sustaining hair stability (Harland et al. 2022). As their count surges, the rigidity of hair fibres intensifies concurrently (Gniadecka et al. 1998). Nevertheless, damage to these bonds may substantially diminish hair rigidity, elevating the risk of hair breakage (Richardson et al. 2017; Yin et al. 2022). In protein cysteinylation, cysteine's role in hair growth has garnered academic attention. Concurrently, in metallothionein (MT), cysteine-enriched residues are integral to cellular metabolism and pivotal in orchestrating zinc homeostasis, notably in its storage and redistribution (Hübner and Haase 2021). Upon oxidation of the zinc-MT complex, zinc is released, potentially acting as a secondary messenger in redox signaling (Gonzalez-Iglesias et al. 2014). This leads to an elevation in free zinc concentration, subsequently activating transcription factors such as nuclear factor erythroid 2-related factor 2 (Nrf2) (Ge et al. 2021; Qin et al. 2021). These factors play a central role in the hair follicle cellular antioxidant response, orchestrating the transcription of various antioxidative enzyme genes (Peterle et al. 2023; Park et al. 2023). Such enzymes mitigate harmful free radicals, fortifying cells against oxidative insults. Within this context, MT serves as an essential nexus between redox and zinc signaling, with cysteine residues playing an instrumental part (Lakha et al. 2023). Enzymes related to cysteine, such as phosphate and tensin homolog deleted on chromosome 10 (PTEN) (Liu et al. 2021) and protein tyrosine phosphatase (PTP) (Persson et al. 2004; Amanakis et al. 2021), encounter activity constraints due to the reversible oxidation of cysteine. Intriguingly, when these enzymes bind with zinc, the oxidation of cysteine residues is inhibited, temporarily reviving their enzymatic activity (Mónico et al. 2021). Proteins like cysteine proteases and PTP, having active sites with cysteine, underscore their significance as targets for zinc and redox signaling modulation. Concurrently, the nexus between zinc and robust hair growth is becoming increasingly clear, with zinc insufficiency potentially hindering hair growth and inducing alopecia (Drake et al. 2023). Existing research reveals that AA patients exhibit notably lower serum zinc levels compared to the healthy populace, underscoring not just the integral role of zinc in hair health but also the pivotal interplay between cysteine and zinc in maintaining hair vitality (Thompson et al. 2017). Cysteine also exerts a pronounced influence on melanin synthesis within hair follicles, especially pivotal in pheomelanin formation (Lee et al. 2021). Additionally, reports indicate that the melanin synthesis process plays a vital role in the pathogenesis of AA (Asz-Sigall et al. 2023). Hence, understanding cysteine's role in hair biology is crucial.

The central concern of this investigation is the potential role of cysteine metabolism in the pathogenesis of AA, a profoundly complex disorder. Although no existing literature directly addresses this concern, potential influencing mechanisms can be extrapolated from the established roles and functions of cysteine within the body.

**Fig. 3:**
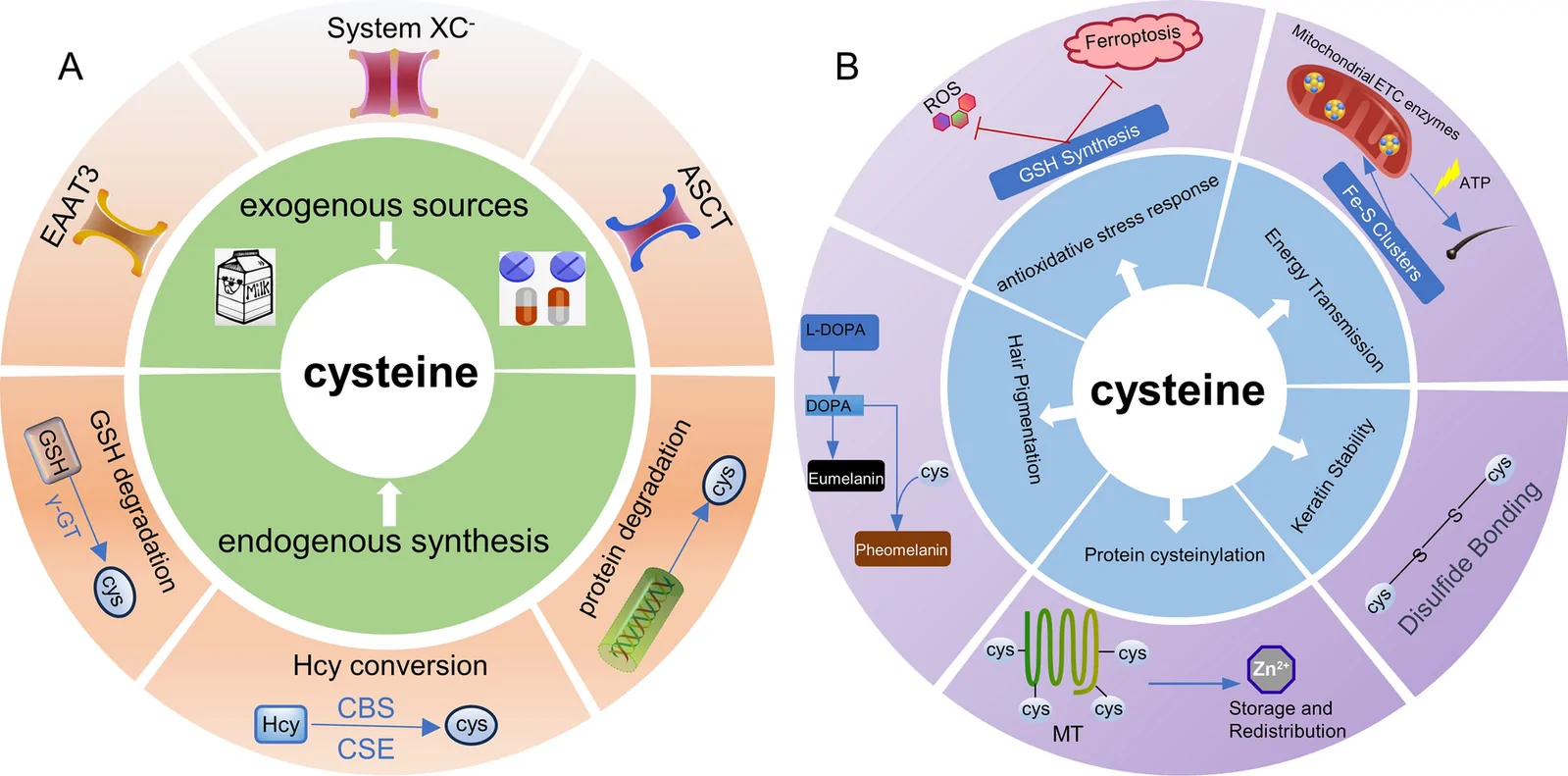
Cysteine Metabolism in Hair Follicles **A** Sources of cysteine in hair follicles primarily originate from both exogenous sources and endogenous synthesis. Exogenous sources are mainly obtained from the diet and drugs through transporters such as System XC-, excitatory amino acid transporter 3 (EAAT3), and alanine–serine–cysteine transporter (ASCT). Endogenous synthesis is predominantly from glutathione (GSH) degradation, homocysteine (Hcy) conversion, and protein breakdown. **B** Cysteine in hair follicles mainly functions in combating oxidative stress through the GSH synthesis, participating in hair pigmentation by pheomelanin synthesis, contributing to Zinc storage and redistribution via protein cysteinylation and metallothionein (MT) synthesis, maintaining the stable structure of hair keratin through disulfide bond formation, and participating in hair follicle energy transmission through the formation of Fe-S Clusters

Cysteine, given its abundance in hair, has a significant positive influence on hair stability. Observable early AA manifestations, including exclamation point hair and hair breakage, suggest that dysregulation in cysteine metabolism may be a significant factor during the initial stages of this condition. One potential mechanism may involve oxidative stress, considering the central role of cysteine in the synthesis of the antioxidant GSH and MT’s critical involvement in zinc signaling pathways. Cysteine metabolic dysregulation could lead to diminished GSH and MT production, intensifying oxidative stress, damaging hair follicles, and potentially causing hair loss. Furthermore, cysteine could affect hair growth by impacting protein synthesis since hair primarily comprises keratin, which is rich in cysteine. If metabolic shifts impact cysteine availability, keratin production could be compromised, affecting hair growth (Fig. 3).

In addition, we delve into the potential roles of cysteine metabolism in various processes during the onset of AA. This narrative underscores the necessity to further dissect the potential influence of cysteine metabolism on AA’s etiology.

## Underlying mechanisms

Building on the introduced roles of cysteine metabolism, it is paramount to delve deeper into the precise mechanisms behind these associations. We will unpack the intricate interactions of cysteine metabolism with autophagy, melanin synthesis, and ferroptosis. Equally significant, the relationship between cysteine metabolism, psychiatric symptoms, and Hcy metabolism in the context of AA will be explored.

### Interplay analysis: cysteine metabolism, autophagy and AA

Cysteine plays a pivotal role in a myriad of cellular functions, spanning from protein synthesis and detoxification to metabolism (Tikhomirova et al. 2023). Its complexity is further accentuated by its association with autophagy, a cellular mechanism responsible for the degradation and recycling of macromolecules and damaged organelles (Mukhopadhyay and Kimmelman 2021). Recent insights from genome-wide association studies (GWAS) have highlighted the significance of autophagy-related genes in AA (Betz et al. 2015). This revelation compels a deeper understanding of their influence on the disease's onset and progression.

Under conditions of nutrient deprivation, cysteine metabolism emerges as an indispensable player in supporting autophagy (Lin et al. 2023). This relationship is orchestrated through intricate feedback loops encompassing coenzyme A (CoA) synthesis, the tricarboxylic acid (TCA) cycle, and amino acid metabolism (Rui 2014). At the cellular level, cysteine demonstrates a notable enhancement of autophagic response during periods of fasting. A particular study indicated that, under oxidative stress conditions, an elevated concentration of cysteine facilitates the biogenesis and maturation of autophagosomes (Jouandin et al. 2022). This suggests that cysteine could modulate autophagy to shield cells from oxidative stress-induced damage. In vivo studies, notably in AA mouse models, have shown that supplementation with NAC (Balansky et al. 2006) combined with autophagy activators (Gund and Christiano 2023) can significantly augment the potential for hair regrowth. This surge in oxidative stress may adversely affect hair follicles, potentially triggering the onset of AA.

To explore these hypotheses in depth, rigorous empirical research is imperative. This might entail modulating cysteine metabolism in both cellular and animal models to study the subsequent impacts on autophagy and the health of hair follicles. As we deepen our grasp on the complex interplay between cysteine metabolism and autophagy, we inch closer to unravelling the potential mechanisms driving the initiation and progression of AA.

### In-depth study: cysteine metabolism and immunity in AA

Cysteine and its metabolic products are increasingly recognized for its potential role in reducing inflammation and modulating immune responses, which are central to the pathogenesis of various autoimmune disorders (Zeinali et al. 2018; Yu et al. 2021; Geng et al. 2023). Supplementation with cysteine alleviates conditions such as mucositis and significantly reduces oxidative stress and systemic inflammation in elderly individuals, suggesting its potential therapeutic benefits in AA (Kumar et al. 2021; Fonseca et al. 2021). *S*-propenyl cysteine, a derivative, can inhibit the NF-κB pathway and activate the Keap1/Nrf2 pathway, mitigating inflammation and oxidative stress, thereby supporting cysteine's role in managing autoimmune disorders (Mo et al. 2020).

In AA, immune cells including CD8+  T cells and TH17 cells play crucial roles. Studies have shown that lowering hydrogen sulfide (H2S) levels by feeding mice a sulfur amino acid-restricted diet increases the ratio of differentiated CD8+ T cells to Tregs (Yue et al. 2023). Additionally, NAC modulates immune responses by reducing the production of Interleukin 17 by TH17 cells, which are vital in autoimmune responses (Duan et al. 2023). This modulation is particularly relevant in AA, where studies have shown that levels of IL-17 are elevated in the serum of patients and positively correlate with the severity of the disease (Loh et al. 2018).

Moreover, cysteine’s anti-inflammatory properties extend to the overall modulation of immune cells. This is particularly relevant in conditions like AA, where immune dysregulation plays a significant role. The ongoing research into cysteine’s impact on oxidative and inflammatory pathways continues to uncover new potential strategies to combat autoimmune disorders like AA, emphasizing cysteine as a promising therapeutic target.

### Confluence exploration: cysteine metabolism, ferroptosis, and AA pathogenesis

Ferroptosis represents a distinct mode of cell death, instigated by lipid peroxidation and markedly reliant on iron ions (Jiang et al. 2021). In this process, cysteine serves as a critical mitigating agent (Yan et al. 2021). Cysteine, entering cells via the System XC-, is oxidized to cystine and participates in GSH generation (Zhou et al. 2020). GSH peroxidase 4 (GPX4) utilizes GSH to reduce lipid peroxides to innocuous lipid alcohols, thereby shielding cells from ferroptotic damage (Seibt et al. 2019). However, upon inhibition of the System XC-, cysteine is denied cellular entry, necessitating an alternative cysteine-dependent mechanism to resist ferroptosis (Tang and Kang 2023). Recent research has revealed a novel mechanism whereby cysteine regulates direct ferroptosis independently of GSH. Mechanistic target of rapamycin complex 1 (mTORC1) detects the concentration of cysteine via the small G protein Recombination activating gene (Rag) and positively regulates the translation and expression of GPX4 protein by phosphorylating eukaryotic initiation factor 4E-binding proteins (4EBPs). The amino-acid transporter solute carrier family 7 member 11(SLC7A11) is a vital component of the System XC-. By reducing SLC7A11, it inhibits the Rag-mTORC1-4EBPs signaling pathway, thereby preventing the entry of cysteine into cells. Therefore, decreased translation and expression of the GPX4 protein regulates ferroptosis (Zhang et al. 2021).

**Fig. 4:**
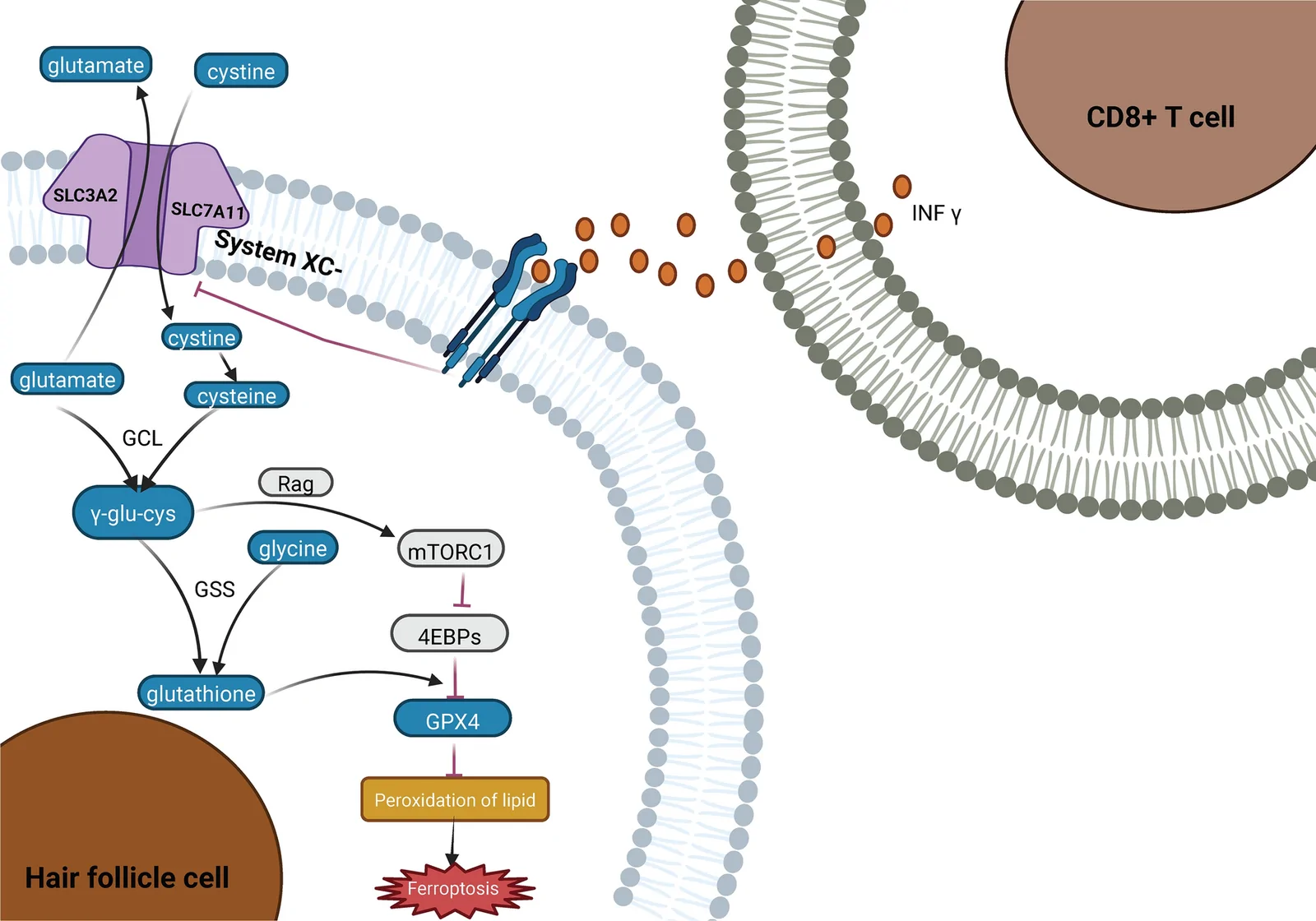
AA Mechanism Hypothesis: Cysteine Metabolism Meets Ferroptosis

In early symptomatic AA patients, it has been observed that CD8+  T cells infiltrate hair follicles substantially (Xu et al. 2023). Current research indicates that T cells can release interferon-γ, which in turn inhibits the System XC-, precipitating a decline in intracellular cysteine levels and triggering ferroptosis (Wang et al. 2019). Moreover, AA patients exhibit elevated blood levels of malondialdehyde (a lipid peroxidation product), potentially causing disorders in cysteine metabolism and heightening the risk of ferroptosis (Cwynar et al. 2018; Sachdeva et al. 2022). Studies reveal that NAC supplementation can effectively lower malondialdehyde concentrations, thereby mitigating oxidative damage to the myocardium during ischemia–reperfusion (Griffiths et al. 2023). Should this mechanism also apply to AA patients, NAC supplementation could potentially rectify impaired cysteine metabolism in these individuals, thereby preventing the compromised expression of GPX4 and the onset of ferroptosis (Fig. 4).

System XC- mediates cysteine uptake in cells by swapping it with glutamate. Its functionality relies heavily on SLC7A11. Interferon-γ (INF-γ) from CD8+  T cells impacts this uptake in hair follicles. Within cells, cystine is converted to cysteine and subsequently to γ-glutamate–cysteine (γ-glu–cys). This pathway aids in the production of GPX4, counteracting lipid peroxidation and deterring ferroptosis. Created with BioRender.com.

These research findings prompt us to propose the hypothesis that both ferroptosis and disturbances in cysteine metabolism might constitute significant mechanisms in the pathogenesis of AA. Furthermore, interventions in cysteine metabolism, such as NAC supplementation, might represent a potential therapeutic approach for AA. This hypothesis offers a fresh vantage point for research and treatment of AA but necessitates further exploration and verification, especially at the molecular mechanism level.

### Hypothetical links examination: psychiatric disorders, hcy metabolism, and AA

Hcy is a precursor to cysteine, centrally involved in the metabolic pathway of methionine (Lazzerini et al. 2007). In conditions marked by the absence of CBS, Hcy tends to accumulate excessively, leading to hyperhomocysteinemia (HHcy) (Poddar 2021). Various factors, including lifestyle habits, nutritional status, and chronic disease states, can precipitate this pathological condition (Poddar 2021). Intriguingly, recent studies indicate that reduction of Hcy levels in HHcy-afflicted mice can alleviate symptoms of facial hair loss (Majtan et al. 2018). HHcy may also induce neuropathological changes, implicating it in the pathogenesis of neurodegenerative disorders (Cordaro et al. 2021). Furthermore, disturbances in Hcy metabolism often coincide with abnormalities in the metabolism of B vitamins, such as B2, B6, B12, and folic acid (Shi et al. 2020). For example, Cardiovascular patients are likely to have been notified by their physicians that their blood levels of Hcy are increased and that they must take folic acid and vitamin B12 to reduce them (Jakubowski 2019). This may be correlated with the observed significantly reduced levels of folic acid and vitamin B12 in AA patients compared to the general population (Thompson et al. 2017).

A recent meta-analysis reveals a higher susceptibility to certain psychiatric disorders, such as depression, in AA patients compared to healthy counterparts (Lee et al. 2019). More notably, Hcy levels show an independent correlation with psychiatric conditions such as depression (Folstein et al. 2007) and obsessive–compulsive disorder (Balandeh et al. 2021), which are both frequently linked to AA (Ohyama 2020; Chou et al. 2022). Interestingly, studies indicate that students experiencing moderate to high levels of anxiety have elevated Hcy levels (Berardis et al. 2020), a finding consistent with observations from Western countries where individuals with depression and older adults also display elevated Hcy levels (Whiteman and Moore 2009). Some research also demonstrated that NAC, known to efficiently reduce plasma levels of Hcy (Hildebrandt et al. 2015), exhibited a favorable clinical response in AA conditions (Balansky et al. 2006). Additionally, NAC has been implicated in offering therapeutic benefits for psychiatric disorders, such as depression (Deepmala et al. 2015). NAC also appears promising in treating trichotillomania, a hair-pulling disorder frequently associated with psychological factors (Kashetsky et al. 2023). However, in a randomized double-blind clinical trial, it was shown that NAC did not demonstrate a statistically significant difference in the treatment of trichotillomania compared to the placebo group (Bloch et al. 2013). This finding suggests that while NAC can influence certain autoimmune and inflammatory responses, its effectiveness may vary significantly across different conditions and symptoms.

Given these complexities, while psychiatric disorders are significantly correlated with AA (Toussi et al. 2021), they might play a secondary role in its pathogenesis. It is hypothesized that the primary role of increasing primary corticotropin-releasing hormone (CRH) might be a more direct driver of hair loss in AA as demonstrated by experimental work (Zhang et al. 2009). Notably, adverse psychiatric symptoms, such as those observed in depression, activate the release of hypothalamic CRH (Gold 2021). Depression is among the most common psychiatric conditions associated with AA (Okhovat et al. 2023), characterized by an excessive release of CRH. Further complicating this pathway, it has been demonstrated that a metabolite of cysteine, hydrogen sulfide (H2S), can regulate CRH release effectively (Kimura 2002). This interplay between cysteine metabolism and psychiatric symptoms may indirectly affect hair structure, thereby adding complexity to the understanding of AA's pathogenesis and underscoring the need for further research in this area.

## Conclusion

Cysteine metabolism plays a crucial role in the pathogenesis of AA, impacting diverse biological pathways ranging from oxidative stress and disturbances in keratin functionality to autophagy, immunity and ferroptosis. While cysteine's influence on these physiological processes is well-documented, recent insights suggest that psychological symptoms commonly observed in AA patients might be secondary complications rather than direct outcomes of cysteine dysregulation. Emerging evidence points to a more significant role of stress-related hormones, such as CRH, which may act as primary drivers in the development of AA. This new understanding could shift the focus of therapeutic interventions towards managing the condition by directly targeting these hormonal pathways alongside regulating cysteine metabolism. However, the research in this area is still in its early stages, and more experimental studies are required to confirm these hypotheses and fully elucidate the complex mechanisms linking cysteine metabolism to AA pathogenesis.

## Data Availability

No data were generated or analyzed during the course of this study.

## Abbreviations

AA: Alopecia Areata
NAC: *N*-acetylcysteine
System XC: Cystine–glutamate transporter exchange transport system
EAAT3: Excitatory amino acid transporter 3
ASCT: Alanine–serine–cysteine transporter
GSH: Glutathione
Hcy: Homocysteine
CBS: Cystathionine-β-synthase
CSE: Cystathionine-γ-lyase
GGT: γ-Glutamyl transpeptidase
ROS: Reactive oxygen species
MT: Metallothionein
Nrf2: Nuclear factor erythroid 2-related factor 2
PTEN: Phosphate and tensin homolog deleted on chromosome 10
PTP: Protein tyrosine phosphatase
CoA: Genome-wide association studies
CoA: Coenzyme A
TCA: Tricarboxylic acid
TRP-1: Tyrosinase-related protein 1
TRP-2: Tyrosinase-related protein 2
GPX4: GSH peroxidase 4
mTORC1: Mechanistic target of rapamycin complex 1
Rag: Recombination activating gene
4EBPs: Eukaryotic initiation factor 4E-binding proteins
γ-glu–cys: Amino-acid transporter solute carrier family 7 member 11(SLC7A11) γ-glutamate–cysteine
INF-γ: Interferon–γ
HHcy: Hyperhomocysteinemia
CRH: Corticotropin–releasing hormone

## Declarations

### Availability of data and materials

All authors gave their consent for publication.
